# A New Approach for the Fabrication of Cytocompatible PLLA-Magnetite Nanoparticle Composite Scaffolds

**DOI:** 10.3390/ijms20194664

**Published:** 2019-09-20

**Authors:** Esperanza Díaz, María Blanca Valle, Sylvie Ribeiro, Senentxu Lanceros‑Mendez, José Manuel Barandiarán

**Affiliations:** 1 Escuela de Ingeniería de Bilbao, Departamento de Ingeniería Minera, Metalúrgica y Ciencia de Materiales, Universidad del País Vasco (UPV/EHU), 48920 Portugalete, Spain; 2 BCMaterials, Basque Centre for Materials, Applications and Nanostructures, UPV/EHU Science Park, 48940 Leioa, Spainjm.barandiaran@ehu.eus (J.M.B.); 3 Facultad de Ciencia y Tecnología, Departamento de Electricidad y Electrónica, University of the Basque Country (UPV/EHU), Sarriena s/n, 48940 Leioa, Spain; mb.valle@ehu.eus; 4 Centro de Física, Universidade do Minho, 4710-057 Braga, Portugal; s.ribeiro@bcmaterials.es; 5 Centre of Molecular and Environmental Biology (CBMA), Universidade do Minho, 4710-057 Braga, Portugal; 6 IKERBASQUE, Basque Foundation for Science, 48013 Bilbao, Spain

**Keywords:** polymer scission, PLLA, magnetite, cytotoxicity, magnetism, in vitro degradation

## Abstract

Magnetic biomimetic scaffolds of poly(L-lactide) (PLLA) and nanoparticles of magnetite (nFe_3_O_4_) are prepared in a wide ratio of compositions by lyophilization for bone regeneration. The magnetic properties, cytotoxicity, and the in vitro degradation of these porous materials are closely studied. The addition of magnetite at 50 °C was found to produce an interaction reaction between the ester groups of the PLLA and the metallic cations of the magnetite, causing the formation of complexes. This fact was confirmed by the analysis of the infrared spectroscopy and the gel permeation chromatography test results. They, respectively, showed a displacement of the absorption bands of the carbonyl group (C=O) of the PLLA and a scission of the polymer chains. The iron from the magnetite acted as a catalyser of the macromolecular scission reaction, which determines the final biomedical applications of the scaffolds—it does so because the reaction shortens the degradation process without appearing to influence its toxicity. None of the samples studied in the tests presented cytotoxicity, even at 70% magnetite concentrations.

## 1. Introduction

Over the past decade, the use of porous matrices in tissue engineering has assumed great importance and, specifically when combined with magnetic materials, they are the subject of in-depth research into their properties. Among the most promising examples of applications from various fields are biomedical applications, and especially bone tissue regeneration [1,2,3]. Regenerative tissue medicine seeks to offer new solutions for the recuperation of both the structure and function of damaged tissues. Scaffolds are porous biomaterials designed to provide an initial mechanical support and a niche for transplanted cells, which promote cell adhesion, proliferation, and differentiation [3]. The time taken for the formation of bone tissue, until the bone recovers full functionality, is quite lengthy (in some cases from six to nine months). For recuperation to follow, various angiogenic proteins some with growth factors must exist in the neighborhood of the scaffold [4,5,6]. The use of growth factors improves the proliferation functions and the differentiation of the cells on the porous matrix, although they have a very short life and quickly lose their functional properties [6,7,8,9]. The principal limitations of the scaffolds are related to the difficulty of controlling cellular differentiation and the processes of angiogenesis, as well as having a stable implant in the pathologically affected area. It is commonplace that bone regeneration is assisted by mechanical stresses originating from the piezoelectric character of hydroxyapatite [10]. Stimulation from an external magnetic field improves the permeability of the cellular membrane, regulates the concentration of calcium ions, and activates channels of cellular signaling, resulting in greater cellular adhesion, proliferation, and differentiation, and therefore an acceleration of bone regeneration [9,11,12].

To obtain magnetic forces from external fields, magnetic nanoparticles must be added to the porous matrices so that they become magnetic, producing osteoconduction through external magnetic fields. For this reason, the study of magnetically charged scaffolds is of great interest in matters of bones. 

Uncoated magnetic nanoparticles tend to agglomerate, are not stable (tending to oxidize) and are harmful for good health. Hence, biodegradable and biocompatible polymers are used to cover them and to form porous matrices that contain them. Polyesters such as PLLA [13], poly(ε-caprolactone) (PCL) [14], poly(Lactide/ε-caprolactone) (PLCL) [15], and poly(Lactide-*co*-Glycolide) (PLGA) [16] among others have been studied, due to their biodegradability and biocompatibility, as well as ease-of-processing with different techniques such as lyophilization [14] and electrospinning [16], etc. 

The fabrication technique for tissue engineering scaffolds depends on the bulk and surface properties of the material and the proposed function of the scaffold. While each method presents distinct advantages and disadvantages, the appropriate technique must be selected to meet the requirements for the specific type of tissue [17,18].

The manufacture technique used in this paper was thermally induced phase separation (lyophilization), a technique that permitted homogeneous dispersion of magnetic nanoparticles (MNPs). Other researchers have manufactured PLLA scaffolds with a lower percentage of nFe_3_O_4_ (5%, 10% and 15% (*w*/*w*)) [2,3]. However, there are not any previous studies at concentrations of 30–70% magnetite in the polymeric matrix. Some authors [19,20] have studied the presence of bio-active particles, such as bioglass, SiO_2_, and nHA, but we are unable to find other reports in the literature of magnetite bonded to the porous matrices that causes a scission of the macromolecular chains and its effects on cytotoxicity, magnetism, and in vitro degradation, and therefore possible biomedical applications.

## 2. Results

### 2.1. Cytotoxicity

**Figure 1:**
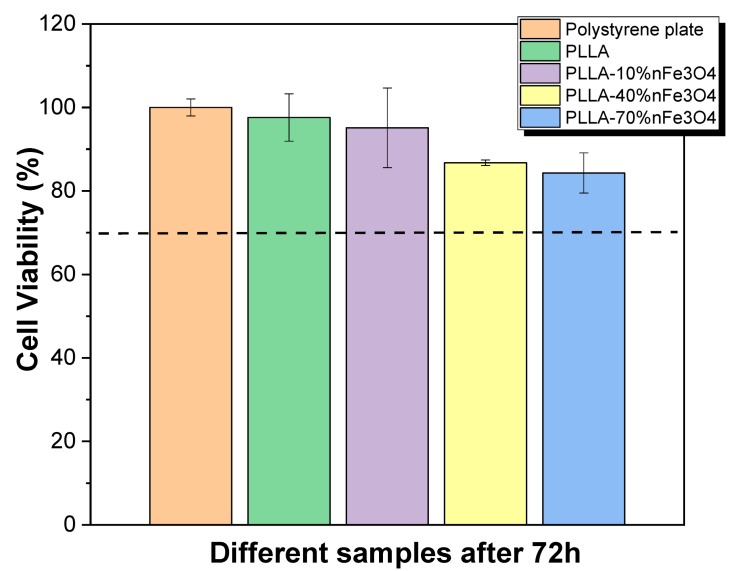
Cell viability of MC3T3-E1 pre-osteoblast cells after 72 h in contact with conditioned media that has been exposed to the different samples during 24 h. The imaginary line is the limit to cell viability after 72 h.

The sample cytotoxicity is an essential parameter for the scaffold to be used in tissue engineering applications. The cytotoxicity of PLLA and PLLA-nFe_3_O_4_ samples was evaluated, according the ISO standard 10993-5, with MC3T3-E1 pre-osteoblast cells. This study was performed after 24 h and 72 h (Figure 1). None of the samples studied in the tests presented cytotoxicity, even at 70% magnetite concentrations.

### 2.2. Magnetic Analysis

Data normalized to the sample mass, determined by an analytical balance down to ± 0.05 mg was within the overall measurement accuracy of 1% for different pieces of the same scaffold, thus indicating the homogeneous distribution of nFe_3_O_4_ in the scaffolds. Correction of the PLLA diamagnetism was irrelevant even at the lowest nFe_3_O_4_ concentrations.

The amount of MNPs of the scaffolds, however, was not exactly nominal, as deduced from the saturation magnetization—that’s why we recalculated the magnetite content in each of the prepared scaffolds using the pure nFe_3_O_4_ as a reference, and disclosed the actual nFe_3_O_4_ content in the scaffolds to be used as a more accurate value for further discussion.

**Figure 2:**
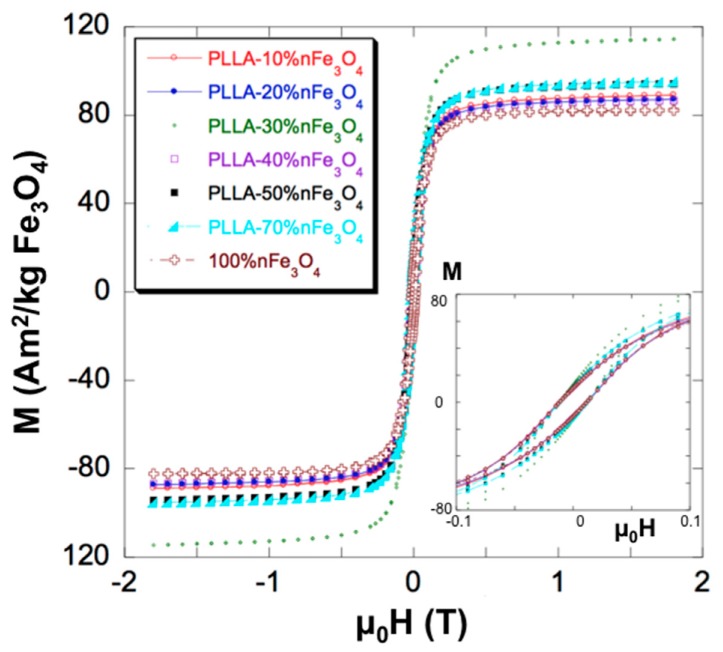
Magnetization curves of pure (100%) nFe_3_O_4_ and PLLA-nFe_3_O_4_ scaffolds normalized to the nominal nFe_3_O_4_ content. The inset shows the initial part of the curves and discloses the coercivity, which is the same for all samples.

Figure 2 shows the hysteresis loops of the PLLA-nFe_3_O_4_ scaffolds at 37 °C. Magnetic parameters, as saturation magnetization measured at 1.5 Tesla (Ms) and coercive field (µ_0_Hc), are listed in Table 1. All the samples studied, Fe_3_O_4_ nanoparticles and PLLA-nFe_3_O_4_, had the coercivity ≈ 12 mT. The true content of nFe_3_O_4_ was recalculated from the experimental value of the magnetization and is also displayed in the table. After such a renormalization, the actual composition range covered by this study is shown to extend up to 80% in weight.

The ferromagnetic (FM) character of the nanoparticles was also clear from the rapid saturation of the magnetization. As the coercivity is the same for all samples, we can conclude that the interaction among the magnetite particles is not important, being well dispersed in the matrix. 

### 2.3. SEM 

**Figure 3:**
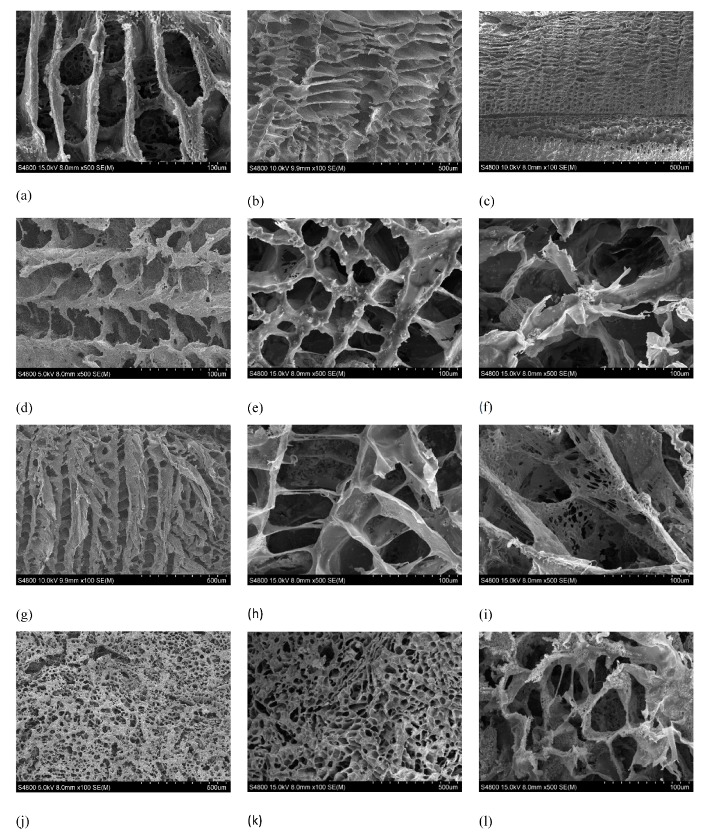
SEM observation of Surface morphology of PLLA and PLLA/nFe_3_O_4_ systems. (**a**) PLLA before in vitro degradation. (**b**) PLLA after in vitro degradation for 8 weeks. (**c**) PLLA after in vitro degradation for 25 weeks. (**d**) PLLA30%nFe_3_O_4_ before in vitro degradation. (**e**) PLLA30%nFe_3_O_4_ after in vitro degradation for 8 weeks. (**f**) PLLA30%nFe_3_O_4_ after in vitro degradation for 25 weeks. (**g**) PLLA50%nFe_3_O_4_ before in vitro degradation. (**h**) PLLA50%nFe_3_O_4_ after in vitro degradation for 8 weeks. (**i**) PLLA50%nFe_3_O_4_ after in vitro degradation for 25 weeks. (**j**) PLLA70%nFe_3_O_4_ before in vitro degradation. (**k**) PLLA70%nFe_3_O_4_ after in vitro degradation for 8 weeks. (**l**) PLLA70%nFe_3_O_4_ after in vitro degradation for 25 weeks.

SEM is a useful tool for studying morphological surface changes in polymeric materials to gain further insight into hydrolytic degradation. SEM images of the different compositions of PLLA and composites of PLLA-nFe_3_O_4_ are showed in the micrographs of Figure 3. All samples studied had a higher porosity and a good level of interconnectivity—this level is of great importance in relation to vascularization and nutrient distribution throughout the tissue. The surfaces of all samples were relatively smooth before and after degradation.

### 2.4. Water Absorption and pH

**Figure 4:**
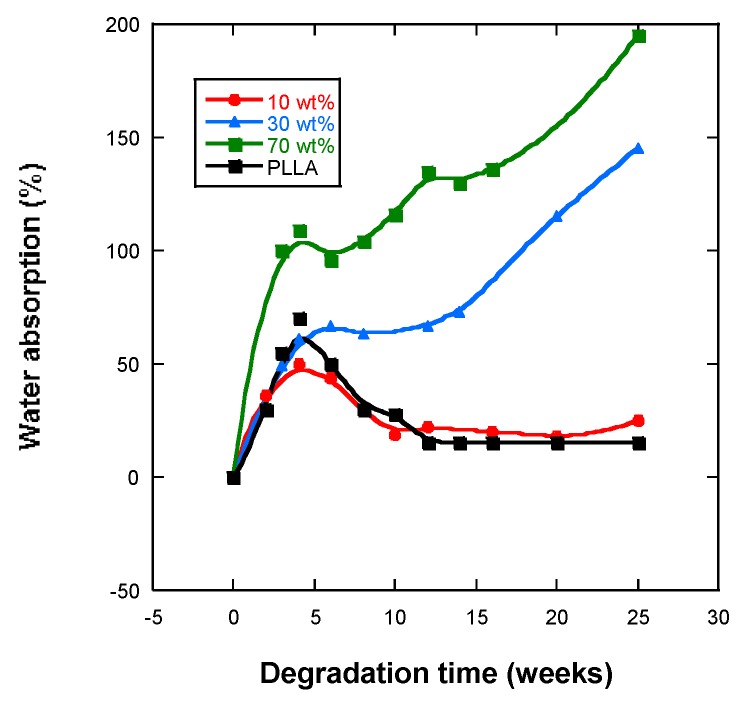
Water absorption by PLLA, PLLA-10%Fe_3_O_4_, PLLA-30%Fe_3_O_4_, PLLA-70%Fe_3_O_4_.

Water absorption into the scaffolds of PLLA is essential for hydrolysis in order to occur within the bulk, otherwise in vitro degradation can only occur at the surface. Figure 4 shows that, over the time of degradation, the water uptake of the different samples increased over four weeks of incubation, and the samples with 30% and 50% magnetite absorbed the highest levels of PBS 150 and 200%, respectively. Scaffolds with 10% and 30% magnetite from the fourth weeks of in vitro degradation decreased the percentage of absorbed water.

**Figure 5:**
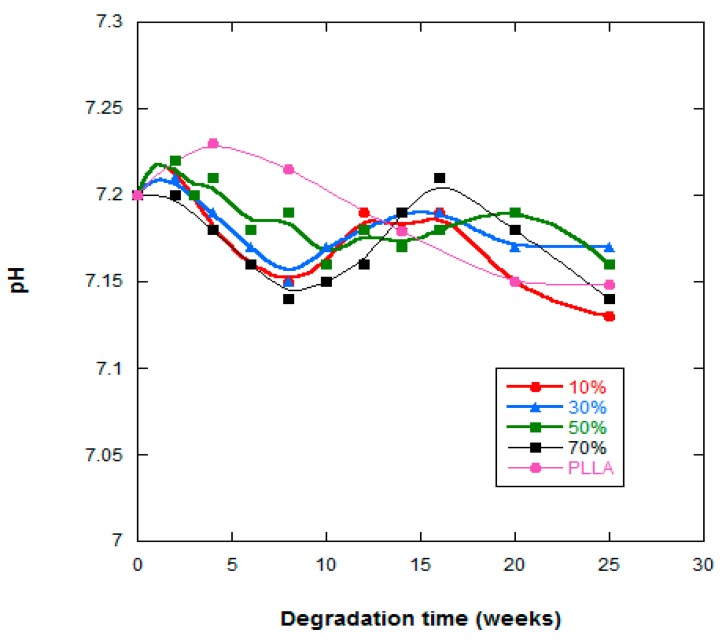
pH of the PBS solution vs. degradation time of PLLA, PLLA-10%Fe_3_O_4_, PLLA-30%Fe_3_O_4_, PLLA-50%Fe_3_O_4_ and PLLA-70%Fe_3_O_4_.

Figure 5 shows the pH changes of the PLLA and PLLA-nFe_3_O_4_ composite scaffolds as a function of in vitro degradation time. With respect to the PLLA system, there was a minimal linear diminution in the value of the pH between 7.22–7.13 (<2%), however the values of the PLLA-nFe_3_O_4_ fluctuated between 7.2–7.09.

### 2.5. Infrared Spectroscopy (FTIR)

**Figure 6:**
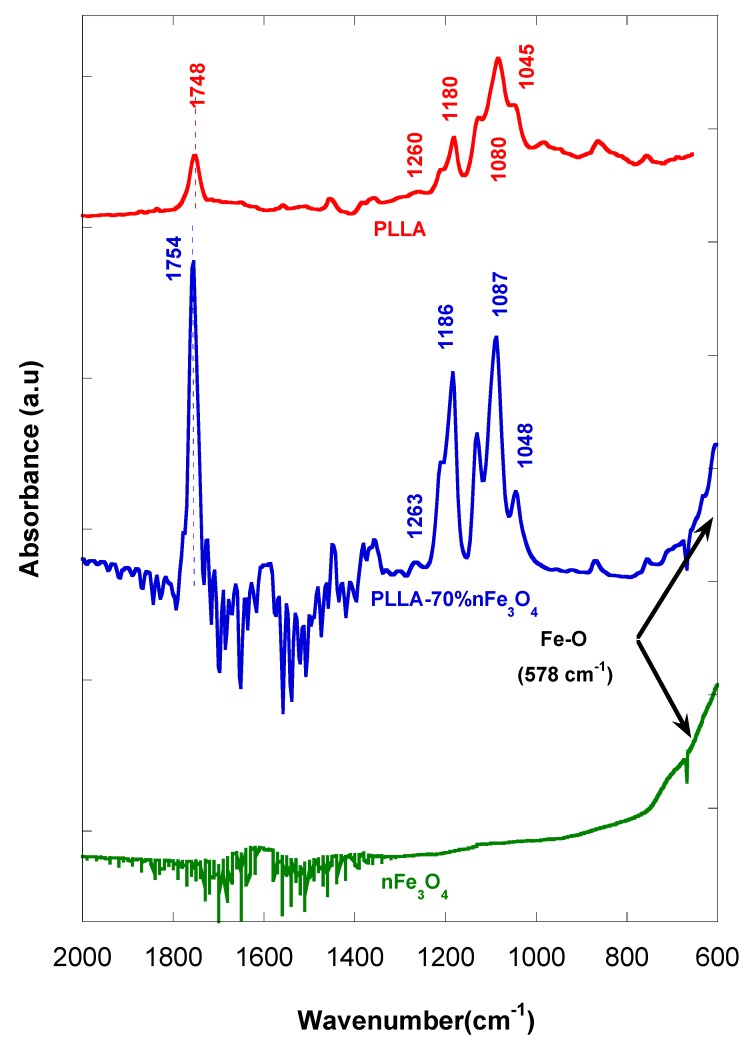
FTIR of nFe_3_O_4_, PLLA and PLLA-70%n nFe_3_O_4_.

In Figure 6, we see that some of the PLLA bands with magnetite additions present some significant shifts with respect to that of PLLA. Some slight changes in position of the bands, at 1754 cm^−1^ (stretching C=O), 1263 cm^−1^ (flexion C=O), 1186 cm^−1^ (stretching -C-O-), and 1087 cm^−1^ (tension -C-O-), are observed. 

**Figure 7:**
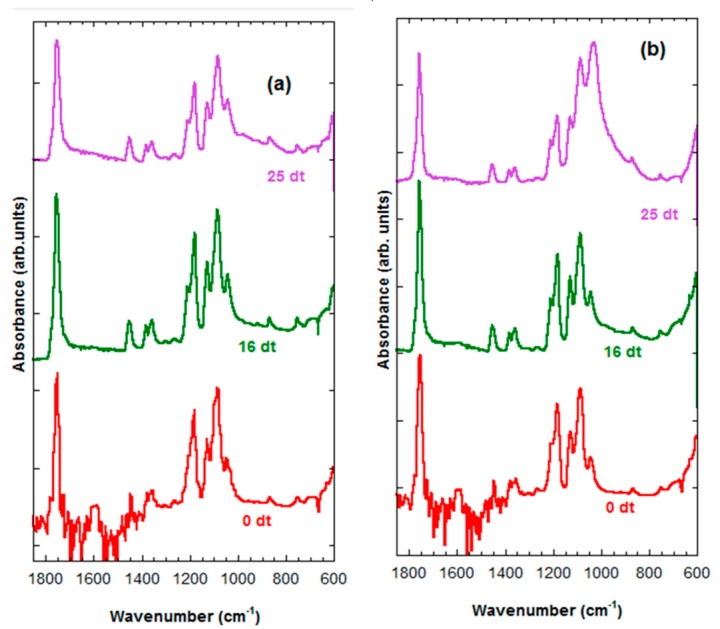
FTIR spectra. (**a**) PLLA-30%nFe_3_O_4_ after various degradation times (0, 16 and 25 weeks), (**b**) PLLA-70%nFe_3_O_4_ after various degradation times (0, 16 and 25 weeks).

In Figure 7a,b, we can see that the FTIR of the PLLA-30%nFe_3_O_4_ and the PLLA-70%nFe_3_O_4_ respectively. The FTIR spectra of PLLA-30%nFe_3_O_4_ remained inalterable before and after 25 weeks of degradation, there are no appreciable changes, as might be expected. The FTIR spectra of PLLA-70%nFe_3_O_4_ presented in the band of 1087 cm^−1^ (tension -C-O-) some shifts after 25 weeks of degradation.

### 2.6. Mass Loss and Weight Loss

The GPC technique determines the changes to the length of macromolecular chains in the polymer caused during the formation and in vitro degradation of the scaffolds. These porous matrices did not have mass loss higher than 3%, which indicates that there had been almost no degradation throughout the incubation period of 25 weeks in PBS. 

In Table 2, we can see that the molecular weight (Mw, Mn) decreased and the polidispersity index (I) of the scaffolds increased as a result of the hydrolysis process in the in vitro degradation, and also by the addition of magnetite for the non-degraded samples.

### 2.7. Differential Scanning Calorimetry (DSC)

In Table 3, the thermal properties of the PLLA-nFe_3_O_4_ system are shown and its evolution against the degradation time. 

It may seem that the fusion peak presents no major changes in its form and position for compositions with a nanoparticle content of less than 50%, which indicates that there have been no major changes in its crystalline structure. Furthermore, it is possible to see that the value of the melting temperature (*T_m_*) remains invariable, at ≈183 °C, the value of the melting enthalpy (Δ*H_m_*) is reduced to 19.9 and to 11.7 J.g^−1^, respectively, contrastable with the value of 41.1 J.g^−1^ for the pure polymer.

## 3. Discussion

Poly-lactones such as poly(L-lactide) are becoming the most commonly used synthetic biodegradable polymer in tissue engineering due to their excellent mechanical property profile, thermoplastic processability, and biological properties such as biocompatibility and biodegradability [21]. Thus, this polymer has been widely used to develop new platforms for tissue engineering, and the introduction of nanoparticles in this polymer can increase their applicability in some areas such as cell mechanotransduction, gene delivery, or thermal stimulation [20,21,22,23,24]. It is reported that nano-magnetite particles, such as Fe_3_O_4_ nanoparticles, increase osteoconductive effect [22,23]. Further, it has been reported that the cytotoxicity of Fe_3_O_4_ nanoparticles depends on their concentration in the polymer matrix [24]. Thus, it has been demonstrated that the introduction of different Fe_3_O_4_ content up to 40% in PLLA matrix leads to not cytotoxic composites [25], however, there are no studies up to 70%. Figure 1 shows that, after 72 h, neat PLLA is not cytotoxic once the cell viability reduction is less than 30%, in concordance with the literature [26,27]. Relative to the PLLA-nFe_3_O_4_ composites, it is observed that these samples are not cytotoxic up to 72 h. From the results obtained, it is verified that the Fe_3_O_4_ are properly encapsulated by the polymer, confirming that these porous nanocomposites can be used as scaffolds for bone tissue engineering under dynamic conditions (applied magnetic field) [28,29]. 

The proper characterization of the materials is important to assess the conditions of their applicability. Thus, Table 1 shows that the coercivity of the initial nFe_3_O_4_ and of all the composite scaffolds was almost the same: µoHc ≈ 12 mT. This coercivity is slightly lower than other values found in the literature, which are about 18–20 mT [30]. The value of the coercivity is high enough to rule out any kind of superparamagnetism, and confirms the ferromagnetic (FM) character of the nFe_3_O_4_, as expected from its particle size (≥30 nm). FM behavior is also supported by the rapid saturation of the magnetization. The strictly constant coercivity, up to the largest nFe_3_O_4_ concentrations, indicates the homogeneous dispersion of the nanoparticles in the PLLA matrix. 

The matrices manufactured with magnetite maintain an internal structure of ladder-like open tubular pores, similar to those manufactured with pure PLLA, a typical morphology formed by the solid-liquid phase separation process [11,12] showing a continuous structure of interconnected pores (Figure 3). Solvent crystallization can induce phase separation when the temperature is lowered to produce a solid–liquid phase separation, the solvent crystallizes and the polymer is expelled from the crystallization front of the solvent, so the polymeric solution undergoes a solid-liquid phase separation [14]. The walls of the pores consisted of PLLA and nFe_3_O_4_ particles, homogeneously distributed in the polymeric PLLA matrix. At low magnification, they reveal the anisotropy of the pores and the presence of defects within the structure. Those defects are due to the solidification of the mixture and occluded air bubbles. The aluminum molds used for their manufacture are very conductive, for which reason the solidification of the mixture started on the walls of the mold, creating cavities towards the interior. This, together with the direction of freezing, favored the appearance of interconnected pores through channels.

We can see in Figure 3 that the addition of nFe_3_O_4_ slightly reduces the average pore size from 100 μm for PLLA to 80, 70 and 60 μm, for the compositions of 30%, 50%, and 70% nFe_3_O_4_, respectively. The introduction of nFe_3_O_4_ particles upset the crystallization of the 1,4 dioxane and modified the growth pattern of the crystals making them more irregular [30,31,32,33]. The result was a more irregular and anisotropic tubular structure, due to the temperature gradient, the parallel channels in the direction of crystallization of the dissolvent [31], and with smaller pores provoking the enlargement of the walls. As the nanoparticle proportions increased, so too did the quantity of piled-up particles and the ruggedness of the surface texture of the pore wall increased.

The internal structure of the original pores was basically maintained in all the samples after 25 weeks of in vitro degradation. However, a slight increase in pore size (≈ 25%) was found, as can be seen in SEM micrographs, which meant that the morphology of the surface was thicker in contrast with the original morphology. At high enlargement, a very porous structure was seen with the fusion of small pores [34]. As from week 16 of degradation, a large quantity of micropores appeared on the walls of the polymeric matrix. The polymeric matrix of the composition 70% nFe_3_O_4_ had a similar appearance to cotton.

Greater crystallinity and greater molecular weight will mean less absorption of PBS, because the diffusion of the solution inside the polymer is easier through the amorphous zones than through the crystalline zones. Hence, the samples under analysis present high levels of swelling (see Figure 4).

Comparing the study samples, it was found that the absorption of PBS increased with the percentage of nanoparticles, nFe_3_O_4_ also increased (Figure 4), and the scaffolds presented lower crystallinity (Table 3) and as expected were also of lower molecular weight (Table 2), which facilitates greater diffusion of the solution with a higher proportion of amorphous parts into the polymer interior [35,36,37]. Water absorption curves show that the introduction of magnetic particles increased their capacity to absorb water, the samples with the highest quantity of nanoparticles absorbing the highest levels of PBS, which has also been observed by other authors [36,37,38]. Such a behavior was expected in a composite polymer material that is hydrophobic, whereas magnetite is very hydrophilic. Water is a degradation medium and its absorption levels are determined by the balance between the oligomer dissolution rate and the water uptake of residual material in the solution.

The variation in pH gives us an idea of the level of acidic residue liberation in the PBS solution and therefore insight into the degradation process (Figure 5). Stabilization of the pH was due to the neutralization of the nanoparticles from the acidic degradation products of this poly-α-hydroxy acid. The PLLA and PLLA-10%nFe_3_O_4_ samples underwent the highest variation in pH, justifiable by the lower quantity of basic and hydrophilic nanoparticles present in its contents.

The spectra of the PLLA-nFe_3_O_4_ (Figure 6) showed typical bands of polymer slightly displaced from 1748 cm^−1^ to 1754 cm^−1^ (stretching C=O) of the PLLA carbonyl groups, interacting with the iron cations on the magnetite surface [39,40,41], from 1260 to 1263 (flexion C=O), from 1180 to 1186 (stretching –C-O-) and from 1080 to 1087 (tension –C-O-) [40], which is an indicator that an interaction exists between the nanoparticles and the polymer.

In addition, in the porous matrices prepared with magnetite, a distinctive peak appeared at 578 cm^−1^ assigned to the vibration of the Fe-O bond of MNPs, not all of which is visible, but it can be estimated at ≈600 cm^−1^ [41,42]. The band became clearer for the scaffolds with higher contents of MNP. 

The use of an ATR (attenuated total reflectance attachment) only allowed us to make a qualitative analysis. In the FTIR of the PLLA-nFe_3_O_4_ samples, we see that the frequencies or stress modes of the carbonyl group appear to be displaced at higher numbers and wave lengths, in the order of 3, 6 and 7 cm^−1^, which suggests the strength of the bond, C=O, has changed. It should be taken into account that the frequency and the constant of force for a particular bond are expressed in equation ν = (K/m)^1/2^/2Π [43] where K is the constant force, m is the mass, and ν is the frequency. In addition, the maximum peak belonging to the carbonyl group of the polymer is displaced in equal proportion to the composition of the metal, as Kurimura et al. demonstrated [44]. It is possible that metallic complexes have been formed through the ester PLLA group and the metallic ions of the magnetite.

In Figure 7a we can see that the FTIR of the PLLA-30%nFe_3_O_4_ remained inalterable before and after 25 weeks of degradation. The IR spectra PLLA-70%nFe_3_O_4_ presented in the band of 1087 cm^−1^ (tension -C-O-) some shifts after 25 weeks of degradation (Figure 7b). In Figure 7a,b no bands were observed of at 1570 cm^−1^ corresponding to the final carboxylic acid groups that should have formed in the process of degradation [45], probably due to the polymer scission process having formed cyclic chains with more compact formations that lead to lower densities of the molecular bonds and greater diffusiveness. 

In a typical process of PLLA degradation, the mass of the samples could be maintained in a first stage, followed by a strong loss of polymer mass [35,46,47] that could indicate the advance of the in vitro degradation process. Our samples barely had a 3% mass loss which indicates that we are in a first stage of the degradation process; these results do not coincide with the works of other authors [46,47], they observed to lose more mass in the PLLA. However, the molecular weight decreased (Table 3). The introduction of nanoparticles in the polymer matrix caused their molecular weight to be reduced by 56.8%, 57.7%, and 78% for samples of PLLA-10%nFe_3_O_4_, PLLA-30%nFe_3_O_4_ and PLLA-50%nFe_3_O_4_ respectively. When the nanoparticles are introduced into the scaffolds, their metallic cations interact with the carbonyl groups of PLLA and provoke macromolecular chain scissions, forming a type of metal-polymer complex that in turn shortens the chains before the onset of the in vitro degradation process [48]. This all agrees with the FTIR results, in which there are stretching and flexural displacements of all the bands of C=O, and stress displacements of -C-O-. The complexes are formed between those parts of the polymer that function as acceptors and the metallic cations that do so as electron donors. The metallic cations of iron, from the sources of magnetite, acted as catalysts provoking macromolecular scission. We can see a lowering of the values of Mw and Mn as the nanoparticle content and the polydispersity value increases, as might have been expected. So, more chains of shorter length and of varying molecular weights are formed. 

During the process of in vitro degradation, the fraction of short chains increased, while the average molar mass by weight diminished. The polydispersity index slightly increased as the molecular weight decreased. The evolution of the properties presented in this study suggested that the polymer was in its first stages of degradation, at least during the 25 weeks of this study. There were no signs of transition towards the second stage of degradation, as indicated by the absence of changes in mass and morphology [49].

In Table 2, the molecular weights of the PLLA-nFe_3_O_4_ scaffolds, once measured, were higher than 50,000 g.mol^−1^, which means that their application in bone fixation devices is feasible [36,50], although the scission of the macromolecular chains provoked by the metallic cations present in the magnetite will diminish its degradation time and therefore limits its applications. It is necessary for the scaffold to have adequate mechanical properties and these depend on the molecular weight during the degradation process.

The crystallization temperature (*T_c_*) of the scaffolds with 20%, 30%, 50%, and 70% nFe_3_O_4_ increased (by 102, 103, 118, and 112 °C respectively) with respect to the pure polymer and the composition with 10%nFe_3_O_4_ (PLLA) (96 °C). In other words, the porous PLLA matrices started to crystallize slower than the pure PLLA and the composition of 10%nFe_3_O_4_. This cycle suggests that the particles of nFe_3_O_4_ do not act as agents of nucleation on the crystallization of the PLLA. Crystallinity (*X_c_*%) dropped from ≈39% in the pure polymer to a value of 11% in the scaffold with a higher quantity of nanoparticles, which dramatically modified the polymer, lowering the degree of crystallinity of the PLLA, complicating crystallization, and reducing the mobility of the chains [41,50].

The value of the polymer crystalline fraction (CF%) considerably increased with the addition of nanoparticles, from 6% for the pure polymer up to ≈73% in the composition with 50% nFe_3_O_4_. CF%, which is linked to reductions in the length of the polymeric chains. If the length of the polymeric chain is reduced by the addition of nanoparticles, the shortest chains will crystallize more easily in the second scan, increasing the value of CF% and reducing crystallinity [37].

The crystalline fraction (CF%) simultaneously increased in almost inverse proportionality with the crystallinity. This is a behavior that might be related to the polymeric chain length. The samples with the lowest crystallinity had shorter polymeric chains where they were affected by degradation and therefore showed a loss of order (i.e., lower crystallinity)—in the second run, these shortened polymeric chains, due to their reduced molecular weight, were able to crystallize, with a consequent increase in CF%. It may, therefore, be affirmed that the samples with the lowest polymer crystallinity and the highest CF correspond to the lowest polymeric chain length [51]. The addition of large amounts of particles (50% and 70%) make the crystallization process difficult although the length of the chains is shorter and that is why we find that for concentrations ≥ 50% of nanoparticles the behavior is different than expected. This behavior also justifies the Tg values obtained. 

The particles act as physical restrictions, reducing the mobility of the polymer chains, producing an increase in Tc and a slight increase in the value of *T_g_* at 56 °C and, in the pure PLLA, at 63 °C for contents of 50%nFe_3_O_4_.

The addition of up to 10%nFe_3_O_4_ had no significant effect on the characteristic temperatures of the scaffolds: *T_g_*, *T_m_*, *T_c_* [44]. Both *T_g_* and *T_c_* underwent greater variation with larger quantities of nanoparticles.

In summary, the samples with a higher content of nanoparticles are of lower crystallinity and have higher enthalpies of fusion. This difference is due to the increased rigidity of the polymeric chains, verified in the crystallinity percentage and the glass transition temperature.

The value of *T_g_* diminished for the degraded samples due to the reduction of molecular weight. During degradation, the long macromolecular chains were broken into other shorter chains, yielding a reduction in molecular weight, results which agreed with those obtained by [45,46]. The compositions of 10% and 30% nFe_3_O_4_, and the *T_g_* increased to higher temperatures and *T_m_* decreased. After 25 weeks of degradation, this may be attributed to weaker mobility of the PLLA polymer chains, due to the presence of crystalline domains [47]. The principal partially degraded chains of PLLA might be the principal factor for these changes of temperature. The crystallinity of these two compositions (10% and 30% of nanoparticles) increased, contrarily to other compositions which diminished. 

The melting temperature (very dependent on the molecular mass) moved to slightly lower values in all the compositions, as well the degradation time increased, and the higher the melting temperature, then the lower the Tg, associated with a lower thickness of the lamellae. 

In conclusion, magnetic biomimetic porous devices of poly(L-lactide) and higher quantities of magnetite nanoparticles appear to be viable for bone regeneration, despite the addition of magnetite that produces macromolecular chain scissions caused by the catalytic action of iron cations and gives rise to PLLA complexes with iron and cyclic polymers. There nevertheless appears to be no influence either on cytotoxicity or on the magnetic properties of the scaffolds manufactured in this way. The shortening of the polymeric chain length reduced their degradation time and will therefore limit their application in subsequent biomedical applications.

## 4. Materials and Methods

### 4.1. Materials

The PLLA of a weight-average molecular weight Mw = 141.940, Mn = 95.680 and the polydispersity index (PI) Mw/Mn = 1.48 were supplied by Purac Biochem, Gorkum, The Netherlands. The magnetite particles were supplied by Sigma-Aldrich, (Darmstadt, Germany). The particle size ≈ 50 μm and density of 2.75 g cm^−3^. Phosphate-buffered saline (PBS) solution purchased from Fluka Analytical (Sigma-Aldrich, Saint Louis, MO, USA), was used as the degradation fluid at a pH of 7.2. 

### 4.2. Fabrication of PLLA/nFe_3_O_4_ of Porous Devices

Scaffolds of PLLA polymer were prepared by freezing and freeze-drying processes (LyoQuest of Telstar, Barcelona, Spain), using 1,4 dioxane (Panreac, Barcelona, Spain) as a solvent. Briefly, a polymer dissolution was prepared at a polymer weight to solvent volume ratio of 2.5% (*w*/*v*), by stirring for 2 h at a temperature of 50 °C after its complete dissolution. Different amounts of magnetite particles were added to the polymer solution, which was then sonicated for 20 min, for dispersal within the polymer matrix and no agglomerates were formed. The resultant solutions yielded highly porous composite scaffolds over seven days, containing different proportions of nFe_3_O_4_ by total mass of polymer (0%, 10%, 20%, 30% and 70%), which were manufactured by lyophilization, using freezing and freeze-drying processes (LyoQuest of Telstar, Barcelona, Spain) for eight days to extract the solvent completely.

### 4.3. In Vitro Degradation

The scaffolds were cut into 0.5 cm^2^ rectangular pieces, weighed, and immersed in test tubes filled with 15 mL of PBS, in order to study the in vitro degradation process. The test tubes were placed in an incubator at 37 °C. The samples were then recovered after 4, 8, 12, 16, 20, 25 weeks and weighed for the determination of water absorption levels. Any pH alteration of the PBS was recorded with a pH meter PCE 228 (PCE Instruments, Pons, Alicante, Spain). The degraded samples were dried to a constant weight in a freeze-dryer and the following equation was used to calculate both the water absorption (Wa%) and the weight loss (W_L_%) percentages:

(1) $$\mathrm{Wa}\%=\frac{W_{w}-W_{r}}{W_{r}}\times 100$$

where, *W_w_* is the weight of the wet sample after removing the surface water and *W_r_* is the weight of a dry sample after degradation.

(2) $$W_{L}\%=\frac{W_{0}-W_{r}}{W_{0}}\times 100$$

where *W*_0_ is the original mass of the scaffold. 

### 4.4. Cytotoxicity

For the in vitro assays, membranes with 0.1 mg.mL^−1^ were cut and sterilized by UV for 2 h before cell seeding (1h each side). After that, the samples were washed five times with PBS solution for 5 min each. The indirect cytotoxicity evaluation of the samples was conducted by adapting the ISO 10993-5 standard test method. 

Briefly, the conditioned media were prepared by immersing the samples in a 24-well tissue culture polystyrene plate with DMEM containing 4.5 g.L^−1^ glucose supplemented with 10% FBS and 1% P/S, at 37 °C in a 95% humidified air containing 5% CO_2_ and incubated for 24 h. 

At the same time, the MC3T3-E1 cells were seeded in the 96-well tissue culture polystyrene plate at the density of 2 × 10^4^ cells.mL^−1^ and incubated for 24 h to allow the cell attachment on the plate. After this time, the culture medium from the 96-well tissue culture polystyrene plate was removed and the as-prepared conditioned media (from the samples) were added to the wells (100 µL). Afterward, the cells were incubated for 72 h and after this time, the evaluation of the cell viability was quantified with MTT assay. A 20% of DMSO was used as a positive control and the cell culture medium was employed as negative control. The MTT assay measures the mitochondrial activity of the cells, which reflects the viable cell number. At this time point (72 h), the medium of every well was removed and fresh medium containing 10% MTT solution (stock solution of 5 mg MTT.mL^−1^ in PBS) was added. After 2 h of incubation, the MTT crystals were dissolved with DMSO and the optical density was measured at 570 nm in a microplate reader (Bioteck). 

All quantitative results were obtained from four replicate samples and controls and were analysed as the average of viability ± standard deviation (SD). 

The percentage of cell viability was calculated with the Equation (3).

(3) $$\mathrm{Cell}\;\mathrm{viability}\left(\%\right)=\frac{\mathrm{absorbance}\;\mathrm{of}\;\mathrm{sample}}{\mathrm{absorbance}\;\mathrm{of}\;\mathrm{negative}\;\mathrm{control}}\times 100$$

### 4.5. Magnetic Analysis

Magnetic measurements were performed in the General Services for Research (SGIker) of the University of the Basque Country, using a vibrating sample magnetometer (VSM) at physiological temperature of 310 K (37 °C) in the magnetic field range of ± 1.8 Tesla (18 kG). Magnetic field resolution was 20µTesla (0.2 G), and moment sensitivity 10^−8^ Am^2^ (10^−5^ emu). The magnetometer was calibrated with pure (99.995%) nickel. The measuring protocol was as follows: the sample was introduced in the VSM at zero magnetic field and the field was increased to 1.8 Tesla in order to saturate the magnetite. Afterwards, measurements were performed while the field was decreased in steps of 0.1 Tesla (1 kOe) down to 0.3 Tesla. Then, the field step was reduced to 0.02 Tesla and the field further decreased to −0.3 Tesla. For the range −0.3 Tesla to −1.8 Tesla, the step was again 0.1 Tesla once the minimum field was attained. The same procedure was followed for the reverse path, i.e., from −1.8 to + 1.8 Tesla to close the loop.

Magnetization data were normalized to the sample mass, determined by an analytical balance down to ± 0.05 mg. In all cases, overall accuracy in the magnetic moment better than 1% was achieved for the magnetic characterization. A pristine PLLA sample was also measured to correct for the diamagnetism of the matrix. Such a correction was irrelevant even at the lowest nFe_3_O_4_ concentrations.

## 5. Differential Scanning Calorimetry (DSC)

Thermal transitions of scaffolds were studied by differential scanning calorimetry (DSC) measurements with a TA Instruments DSC Q-200. Samples (≈5 mg) of porous matrices were heated from 0 °C to 200 °C at a rate of 10 °C.min^−1^ (first run). Following that treatment, the samples were quenched, and a third scan was conducted from 0 °C to 200 °C, in which the thermal transitions of the material were determined. The first scan was used to record the melting temperature and the fusion heat. The melting temperature, Tm, was noted as the maximum value of the melting peaks, and the mid-point temperature of the glass transition was determined as the glass-transition temperature, Tg. Crystallinity, Xc, was calculated with the Equation (3):

X_c_ (%) = 100 × (∆H_melt_ − ∆H_crystallization_)/∆H_100%_ (4)

where ∆H_melt_ (J g^−1^ of the crystalline polymer) is the enthalpy of fusion of the specimen and ∆H_100%_ is the enthalpy of fusion of a 100% crystalline polymer—which, for PCL, was ΔH_m_^0^ = 93 J/g J.g^−1^. The crystallizable fraction (CF%) of the samples was calculated with the Equation (2):

CF (%) = 100 × (∆H_c_/(∆H_m_) (5)

## 6. Infrared Spectroscopy (FTIR)

Infrared spectra of these scaffolds were recorded on a ThermoNicolet AVATAR 370 Fourier-transform infrared spectrophotometer (FTIR, Thermo Electron Corporation, MA, USA) equipped with an attenuated total reflectance attachment, a, and a ZnSe crystal. Spectra were taken with a resolution of 4 cm^−1^ and were averaged over 32 scans. The absorbance of all the studied samples was within the absorbance range in which the Lambert–Beer law is obeyed. 

## 7. Gel Permeation Chromatography (GPC)

The molecular weight (g.mol^−1^) of the samples was determined by GPC using a Perkin Elmer 200, Waltham, MA, USA in tetrahydrofuran (THF). Calibration adhered to polystyrene standards at a flow rate of 1ml.min^−1^. Three replications have been made for each sample.

## Figures and Tables

**Table 1:** Saturation Magnetization (Ms) normalized to the nominal content of nFe_3_O_4_, Coercivity (μ_0_ Hc) and true nFe_3_O_4_ content recalculated from the saturation magnetization.

wt % nFe_3_O_4_ (Nominal)	Ms (Am^2^/kg nFe_3_O_4_)	μ_0_ Hc (mT)	wt % nFe_3_O_4_ (Recalculated)
100 *	82.05	11.87	-
10	88.70	12.05	10.8
20	86.85	11.75	21.2
30	114.04	11.75	41.7
40	86.30	11.67	42
50	94	11.62	57
70	94.82	11.83	80.5

(*) Used as a reference.

**Table 2:** Molecular weight: weight average (Mw), number average (Mn), and polydispersity index I of the PLLA- nFe_3_O_4_ system.

Sample	Degradation Time(Weeks)	Mw	%Mw	Mn	I
**PLLA**	0	144,221		104,042	1.386
	15	50,365	65.07	27,894	1.800
	25	31,420	78.21	20,123	1.958
**PLLA-10%nFe_3_O_4_**	0	86,087	40.30	55,270	1.558
	15	70,655	48.99	36,196	1.952
	25	51,040	64.60	28,758	1.775
**PLLA-30%nFe_3_O_4_**	0	62,280	56.81	35,695	1.745
	15	32,867	77.21	22,501	1.461
	25	50,367	65.10	20,911	2.409
**PLLA-50%nFe_3_O_4_**	0	61,018	57.69	33,257	1.835
	14	39,878	72.35	24,554	1.624
	25	31,721	78.00	14,722	2.155

**Table 3:** Parameters obtained by DSC on the PLLA-nFe_3_O_4_ scaffolds: Tm = melting temperature (°C), ΔH_m_ = melting enthalpy (kJ/kg), Tc = crystallization temperature (°C), ΔH_c_ = crystallization enthalpy (kJ/kg), Tg = glass transition temperature (°C), Xc = crystalline fraction (%) and CF% = crystalline fraction.

PLLA/%nFe_3_O_4_ t_deg_(weeks)	First Scan				Second Scan			X_c_ % ^a^	CF% ^b^	X_cc_% ^c^
	T_m1_ (°C)	ΔH_m1_ (J/g)	T_cc_ (°C)	ΔH_cc_ (J/g)	T_g_ (°C)	T_c_ (°C)	ΔH_c_ (J/g)			
**0%, 0dt**	184	41.1	76	4.7	56	96	2.3	39	6	39
**8dt**	183	42	76.5	4	57	96	2.5	41	6	39
**25dt**	184	42.3	76.5	4	56.5	96	2.8	41	6.6	39.3
**10%, 0dt**	183	37.9	76	4	57.5	96	4.1	36	11	40.5
**4dt**	180	38.6	85.5	4.0	57	98.5	12.5	37	32	37
**8dt**	180	43.5	85	6.9	58	96	4.2	39	10	44
**14dt**	182	39.8			57	96	4.6	43	12	47.5
**20dt**	180	40.3	72.5	4.1	58.5	98	4.8	39	12	43
**20%, 0dt**	183	37.1		3.9	59	102	19.7	36	53	45
**30%, 0dt**	184	35.5	76.5	1.8	61	103	19.3	36	54	52
**4dt**	183	35.6	76.5	2.4	59	101	14.5	36	41	51
**8dt**	183	36.5	77	1.3	58.5	102	17	38	47	54
**12dt**	183	37.9			58	100	8.7	41	23	58
**16dt**	183	37.5			59	100	6.9	40	18	57
**20dt**	183	37.9			60	98	4.6	41	12	58
**25dt**	182	40			62	100	7.6	43	19	61
**50%, 0dt**	183	19.9	76.4	2.5	63	118	14.6	19	73	37
**4dt**	182	21.4			59	104	9.7	23	45	46
**8dt**	182	19.1			58	101	7.6	21	40	41
**25dt**	180	16.6	80	0.7	59	100	5.3	17	32	34
**70%, 0dt**	183	11.7	72	1.2	62	112	6.8	11	58	38
**4dt**	182	11.1	72	0.5	58	104	4.8	11	43	38
**8dt**	183	10.2	75	0.7	57	112	4.3	10	42	34
**12dt**	183	9			59	106	3.5	10	39	32
**16dt**	182	9.1			50	102	2.7	10	30	33
**20dt**	182	9	75	1.1	58	102	2.6	9	29	28
**25dt**	181	9			57	103	3.2	10	36	32

X_c_ % = 100((ΔH_m1_ − ΔH_cc_)/ΔH_m_^0^); ΔH_m_^0^ = 93 J/g (b) CF % = 100(ΔH_c_/ΔH_m1_). (c) X_corr_ % = 100(ΔH_m1_/W_PLLA_ΔHm^0^); W_PLLA_ polymer fraction.

## Abbreviations

PLLA	poly(L-lactide)
Fe_3_O_4_	magnetite
PCL	poly(ε-caprolactone)
PLCL	poly(ε-caprolactone)
PLGA	poly(Lactide-*co*-Glycolide)
MNPs	magnetic nanoparticles
MC3T3-E1	preosteoblast cells
PBS	phosphate-buffered saline
DMEM	Dulbbecco’s Modified Eagle’s Medium-high glucose (DMEM)
SEM	Scanning electron microscopy
DSC	Differential scanning calorimetry
*FTIR*	Fourier-Transform Infrared Spectroscopy
VSM	Vibrating Sample Magnetometer
GPC	Gel permeation chromatography
MTT	Colorimetric assay for assessing cell metabolic activity

## References

[1] Liu Y., Lu Y., Tian X., Cui G., Zhao Y., Yang Q., Yu S., Xing G., Zhang B. (2009). Segmental bone regeneration using an rhBMP-2-loaded gelatin/nanohydroxyapatite/fibrin scaffold in a rabbit model. Biomaterials.

[2] Whitaker M.J., Quirk R.A., Howdle S.M., Shakesheff K.M. (2001). Growth factor release from tissue engineering scaffolds. J. Pharm. Pharmacol..

[3] Glowacki J. (1998). Angiogenesis in fracture repair. Clin. Orthop. Relat. Res..

[4] Liu J.M., Zhang J., Zhang X., Hlavaty K.A., Ricci C.F., Leonard J.N., Shea L.D., Gower R.M. (2016). Transforming growth factor-beta 1 delivery from microporous scaffolds decreases inflammation post-implant and enhances function of transplanted islets. Biomaterials.

[5] Milkiewicz M., Ispanovic E., Doyle J.L., Haas T.L. (2006). Regulators of angiogenesis and strategies for their therapeutic manipulation. Int. J. Biochem. Cell. Biol..

[6] Causa F., Netti P.A., Ambrosio L.A. (2007). multi-functional scaffold for tissue regeneration: The need to engineer a tissue analogue. Biomaterials.

[7] Azeem A., Marani L., Fuller K., Spanoudes K., Pandit A., Zeugolis D.I. (2016). Influence of Nonsulfated Polysaccharides on the Properties of Electrospun Poly(lactic-co-glycolic acid) Fibers. ACS Biomater. Sci. Eng..

[8] Huang H.M., Lee S.Y., Yao W.C., Lin C.T., Yeh C.Y. (2006). Static magnetic fields up-regulate osteoblast maturity by affecting local differentiation factors. Clin. Orthop. Relat. Res..

[9] WãJcik-Piotrowicz K., Kaszuba-ZwoiåSka J., Rokita E., Thor P. (2016). Cell viability modulation through changes of Ca^2+^-dependent signalling pathways. Prog. Biophys. Mol. Biol..

[10] Zhang K., Wang S., Zhou C., Cheng L., Gao X., Xie X., Sun J., Wang H., Weir M.D., Reynolds M.A. (2018). Advanced smart biomaterials and constructs for hard tissue engineering and regeneration. Bone Res..

[11] Zhu Y., Yang Q., Yang M., Zhan X., Lan F., He J., Gu Z., Wu Y. (2017). Protein Corona of Magnetic Hydroxyapatite Scaffold Improves Cell Proliferation via Activation of Mitogen-Activated Protein Kinase Signaling Pathway. ACS Nano..

[12] Arjmand M., Ardeshirylajimi A., Maghsoudi H., Azadian E. (2017). Osteogenic differentiation Potential of Mesenchymal Stem Cells cultured on Nanofibrous Scaffold Improved in the Presence of Pulsed Electromagnetic Field. J. Cell. Physiol..

[13] Yang F., Murugan R., Ramakrishna S., Wang X., Ma Y.X., Wang S. (2004). Fabrication of nano-estructured porous PLLA scaffolds intended for nerve tissue engineering. Biomaterials.

[14] Díaz E., Sandonis I., Valle M.B. (2014). In vitro degradation of Poly(caprolactone)/nHA composites. J. Nanomater..

[15] Jeong S.I., Kwon J.H., Lim J.I., Cho S.W., Jung Y., Sung W.J. (2005). Mechano-active tissue engineering of vascular smooth muscle using pulsatile perfusion bioreactors and elastic PLCL scaffolds. Biomaterials.

[16] Díaz E., Ibañez I., Puerto I. (2015). Encyclopedia of Biomedical Polymers and Polymeric Biomaterials, 11 Volume Set.

[17] Baek C.H., Ko Y.J. (2006). Characteristics of tissue-engineered cartilage on macroporous biodegradable PLGA scaffold. Laryngoscope.

[18] Huang D.M., Hsiaon J.K., Chen Y.C., Chien L.Y., Yao M., Yin-Kai C.H., Bor-Sheng K., Szu-Chun H., Lin-Ai T., Hui-Ying C.H. (2009). The promotion of human mesenchymal stem cell proliferation by superparamagnetic iron oxide nanoparticles. Biomaterials.

[19] Chrissafis K., Antoniadis G., Parskevopoulos K.M., Vassiliou A., Bikiaris D.N. (2007). Comparative study of the effect of different nanoparticles on the mechanical properties and thermal degradation mechanism of in situ prepared poly (ε-caprolactone) nanocomposites. Compos. Sci. Technol..

[20] Li J.L., Zheng W., Li L., Zheng Y., Lou X. (2009). Thermal degradation kinetics of g-HA/PLA composite. Thermochim. Acta.

[21] Gupta B., Revagade N., Hilborn J. (2007). Poly(lactic acid) fiber: An overview. Prog. Polym. Sci..

[22] Wu Y., Jiang W., Wen X., He B., Zeng X., Wang G., Gu Z. (2010). A novel calcium phosphate ceramic-magnetic nanoparticle composite as a potential bone substitute. Biomed. Mater..

[23] Shan D., Shi Y., Duan S., Wei Y., Cai Q., Yang X. (2013). Electrospun magnetic poly(l-lactide) (PLLA) nanofibers by incorporating PLLA-stabilized Fe_3_O_4_ nanoparticles. Mater. Sci. Eng. C.

[24] Zhang H., Xia J., Pang X., Zhao M., Wang B., Yang L., Wan H., Wu J., Fu S. (2017). Magnetic nanoparticle-loaded electrospun polymeric nanofibers for tissue engineering. Mater. Sci. Eng. C.

[25] Lai W.Y., Feng S.W., Chan Y.H., Chang W.J., Wang H.T., Huang H.M. (2018). In vivo investigation into effectiveness of Fe_3_O_4_/PLLA nanofibers for bone tissue engineering applications. Polymers.

[26] Areias A.C., Ribeiro C., Sencadas V., García-Giralt N., Diez-Perez A., Ribelles J.G., Lanceros-Méndez S. (2012). Influence of crystallinity and fiber orientation on hydrophobicity and biological response of poly(l-lactide) electrospun mats. Soft Matter.

[27] Ribeiro C., Sencadas V., Areias A.C., Gama F.M., Lanceros-Méndez S. (2015). Surface roughness dependent osteoblast and fibroblast response on poly(L-lactide) films and electrospun membranes. J. Biomed. Mater. Res. A.

[28] Ribeiro C., Sencadas V., Correia D.M., Lanceros-Méndez S. (2015). Piezoelectric polymers as biomaterials for tissue engineeting applications. Colloids Surf. B.

[29] Ribeiro C., Correia V., Martins P., Gama F.M., Lanceros-Mendez S. (2016). Piezo-and magnetoelectric polymers as biomaterials for novel tissue engineering strategies. MRS Advances..

[30] Rescignano N., Gonzalez-Alfaro Y., Fantechi E., Mannini M., Innocenti C., Ruiz-Hitzky E., Kenny J.M., Armentano I. (2015). Design, development and characterization of a nanomagnetic system based on iron oxide nanoparticles encapsulated in PLLA-nanospheres. Eur. Polym. J..

[31] Schugens C., Maquet V., Grandfils C., Jerome R., Teyssie P. (1996). Biodegradable and macroporous polylactide implants for cell transplantation. 1.Preparation of macroporous polylactide supports by solid-liquid phase separation. Polymer.

[32] Zhang R., Ma P.X. (1999). Porous poly(l-lactic acid)/apatite composites created by biomimetic process. J. Biomed. Mater. Res..

[33] Nam Y.S., Park T.G. (1999). Porous biodegradable polymeric scaffolds prepared by thermally induced phase separation. J. Biomed. Mater. Res..

[34] Schugens C., Maquet V., Grandfils C., Jerome R., Teyssie P. (1996). Polylactide macroporous biodegradable implants for cell transplantation. II. Preparation of polylactide foams by liquid-liquid phase separation. J. Biomed. Mater. Res..

[35] Zhang Q., Jiang Y., Zhang Z., Ye W., Lang M. (2012). Effect of porosity on long-term degradation of poly (ε-caprolactone) scaffolds and their celular response. Polym. Degrad. Stab..

[36] Zuluaga F. (2013). Algunas aplicaciones del ácido poli-L-láctico. Rev. Acad. Colomb. Cienc..

[37] Díaz E., Sandonis I., Puerto I., Ibañez I. (2014). In Vitro Degradation of PLLA/ nHA Composite Scaffolds. Polym. Eng. Sci..

[38] Huang J., Xiong J., Liu J., Zhu W., Wang D. (2013). Investigation of the In Vitro Degradation of a Novel Polylactide/Nanohydroxyapatite Composite for Artificial Bone. J. Nanomater..

[39] Lafisco M., Palazzo B., Ito T., Otsuka M., Senna M., Delgado-Lopez J.M., Gomez-Morales J., Tampieri A., Prat M., Rimondini L. (2012). Preparation of core–shell poly(L-lactic) acid-nanocrystalline apatite hollow microspheres for bone repairing applications. J. Mater. Sci. Mater. Med..

[40] Kister G., Cassanas G., Vert M. (1998). Effects of morphology, conformation and configuration on the IR and Raman spectra of various poly(lactic acid)s. Polymer.

[41] Nan A., Turcu R., Craciunescu I., Pana O., Scharf H., Liebscher J. (2009). Microwave-assited graft polymerization of ε-caprolactone onto magnetite. Polym. Sci. Part A Polym. Chem..

[42] Yuan W., Yuan J., Zhou L., Wu S., Hong X. (2010). Fe_3_O_4_@ poly (2-hydroxyethyl methacrylate)-graft-poly (ε-caprolactone) magnetic nanoparticles with branched brush polymeric shell. Polymers.

[43] Atkins P., de Paula J. (2008). Química-Física.

[44] Kurimura Y., Tsuchida E., Kaneko M. (1971). Preparation and properties of some water-soluble cobalt (III)–poly-4-vinylpyridine complexes. J. Polym. Sci..

[45] Partini M., Pantani R. (2007). FTIR analysis of hydrolysis in aliphatic polyesters. Polym. Degrad. Stab..

[46] Lam C.X.F., Hutmacher D.W., Schantz J.T., Woodruff M.A., Teoh S.H. (2008). Evaluation of polycaprolactone scaffold degradation for 6 months in vitro and in vivo. J. Biomed. Mater. Res. A.

[47] Yeo A., Rai B., Sju E., Cheong J., Teoh S. (2008). The degradation profile of novel, bioresorbable PCL-TCP scaffolds: An in vitro and in vivo study. J. Biomed. Mater. Res. A.

[48] Díaz E., Valenciano R., Katime I.A. (2004). Study of complexes of poly(vinyl pyrrolidone) with copper and cobalt on solid state. J. Appl. Polym. Sci..

[49] Deplaine H., Acosta-Santamaría V.A., Vidaurre A., Gómez-Ribelles J.L., Doblaré M., Ochoa I., Gallego-Ferrer G. (2014). Evolution of the properties of a poly(l-lactic acid) scaffold with double porosity during in vitro degradation in a phosphate-buffered saline solution. J. Appl. Polym. Sci..

[50] Shyam-Roy S. (2003). Synthesis of Biodegradable Poly (Lactic Acid) Polymers. Ph.D. Thesis.

[51] Taddei P., Di Foggia M., Causa I., Ambrosio C. (2006). In vitro bioactivity of poly(caprolactone)-apatite (PCL-AP) scaffolds for bone tissue engineering: The influence of the PCL-AP ratio. Int. J. Artif. Organs.

